# ACP-DL: A Deep Learning Long Short-Term Memory Model to Predict Anticancer Peptides Using High-Efficiency Feature Representation

**DOI:** 10.1016/j.omtn.2019.04.025

**Published:** 2019-05-10

**Authors:** Hai-Cheng Yi, Zhu-Hong You, Xi Zhou, Li Cheng, Xiao Li, Tong-Hai Jiang, Zhan-Heng Chen

**Affiliations:** 1 The Xinjiang Technical Institute of Physics and Chemistry, Chinese Academy of Sciences, Urumqi 830011, China; 2 University of Chinese Academy of Sciences, Beijing 100049, China

**Keywords:** anticancer peptides, long short-term memory, deep learning, binary profile feature, *k*-mer sparse matrix

## Abstract

Cancer is a well-known killer of human beings, which has led to countless deaths and misery. Anticancer peptides open a promising perspective for cancer treatment, and they have various attractive advantages. Conventional wet experiments are expensive and inefficient for finding and identifying novel anticancer peptides. There is an urgent need to develop a novel computational method to predict novel anticancer peptides. In this study, we propose a deep learning long short-term memory (LSTM) neural network model, ACP-DL, to effectively predict novel anticancer peptides. More specifically, to fully exploit peptide sequence information, we developed an efficient feature representation approach by integrating binary profile feature and *k*-mer sparse matrix of the reduced amino acid alphabet. Then we implemented a deep LSTM model to automatically learn how to identify anticancer peptides and non-anticancer peptides. To our knowledge, this is the first time that the deep LSTM model has been applied to predict anticancer peptides. It was demonstrated by cross-validation experiments that the proposed ACP-DL remarkably outperformed other comparison methods with high accuracy and satisfied specificity on benchmark datasets. In addition, we also contributed two new anticancer peptides benchmark datasets, ACP740 and ACP240, in this work. The source code and datasets are available at https://github.com/haichengyi/ACP-DL.

## Introduction

Cancer is one of the most devastating killers of human beings, accounting for millions of deaths around the world each year.^1^, ^2^ Conventional physical and chemical methods, including targeted therapy, chemotherapy, and radiation therapy, remain the principle modes to treat cancer, which focus on killing the diseased cells, but normal cells are also adversely affected.^3^, ^4^ More obviously, these treatments are expensive and inefficient, which means there is an urgent need to develop novel efficient measures to solve this deadly disease.^5^ The discovery of anticancer peptides (ACPs), a kind of short peptide generally with a length less than 50 amino acids and most of which are derived from antimicrobial peptides (AMPs), often cationic in nature, has led to the emergence of a novel alternative therapy to treat cancer.

ACPs open a promising perspective for cancer treatment, and they have various attractive advantages,^6^, ^7^ including high specificity, ease of synthesis and modification, low production cost, and so on.^8^ ACPs could interact with the anionic cell membrane components of only cancer cells, and, for this reason, they can selectively kill cancer cells with almost no harmful effect on normal cells.^4^, ^9^ In addition, few ACPs, e.g., cell-penetrating peptides or peptide drugs, inhibit the cell cycle or any other functionality. Thus, they are safer than traditional broad-spectrum drugs, which have become the most competitive choice as therapeutics compared to small molecules and antibodies. In recent years, ACP therapeutics have been extensively explored and used to fight various tumor types across different phases of preclinical and clinical trials.^10^, ^11^, ^12^, ^13^, ^14^ However, only a few of them can eventually be employed for clinical treatment. Furthermore, it’s time-consuming, expensive, and lab-limited to identify potential new ACPs by experiment.

With the huge therapeutic importance of ACPs, there is an urgent need to develop highly efficient prediction techniques. Some notable research has been reported in the prediction of ACPs.^15^ Tyagi et al.^16^ developed a support vector machine (SVM) model using amino acid composition (AAC) and dipeptide composition as input features on experimentally confirmed anticancer peptides and random peptides derived from the Swiss-Prot database. Hajisharifi et al.^17^ also reported an SVM model using Chou’s^18^, ^19^ pseudo AAC (PseAAC) and the local alignment kernel-based method. Vijayakumar and Ptv^20^ proposed that, between ACPs and non-ACPs, there was no significant difference in AAC observed. Also, they presented a novel encoding measure, which achieved better predictive performance than AAC-based features, considering both compositional information and centroidal, distributional measures of amino acids. Shortly afterward, based on the optimal g-gap dipeptide components, by exploring the correlation between long-range residues and sequence-order effects, Chen et al.^21^ described iACP, which exhibited the best predictive performance at that time. More recently, Wei et al.^22^ developed a sequence-based predictor called ACPred-FL, which uses two-step feature selection and seven different feature representation methods.

According to the cognition of the short length of ACPs, it’s difficult to exploit the efficient features of many mature feature representation methods, which are widely used on protein sequences.^23^ With the rapid growth of the number of ACPs that has been identified experimentally, by machine learning, and by bioinformatics research,^24^, ^25^, ^26^, ^27^, ^28^, ^29^, ^30^, ^31^, ^32^, ^33^, ^34^, ^35^, ^36^, ^37^, ^38^, ^39^, ^40^ the computational prediction methods of ACPs still need further development.

In this study, we proposed a deep learning long short-term memory (LSTM) neural network model to predict anticancer peptides, which we named ACP-DL. The efficient features exploited from peptides sequences are fed as input to train the LSTM model. More specifically, peptide sequences are transformed by *k*-mer sparse matrix of the reduced amino acid alphabet,^41^, ^42^ which is a 2D matrix, and retained almost complete sequence order and amino acid component details. Meanwhile, peptide sequence are also converted by a binary profile feature,^43^ which can be regarded as one-hot encoding of categorical variables and has been suggested to be an efficient feature extraction technique.^16^, ^22^ Finally, these features are fed into our LSTM model to predict new anticancer peptides.

**Figure 1:**
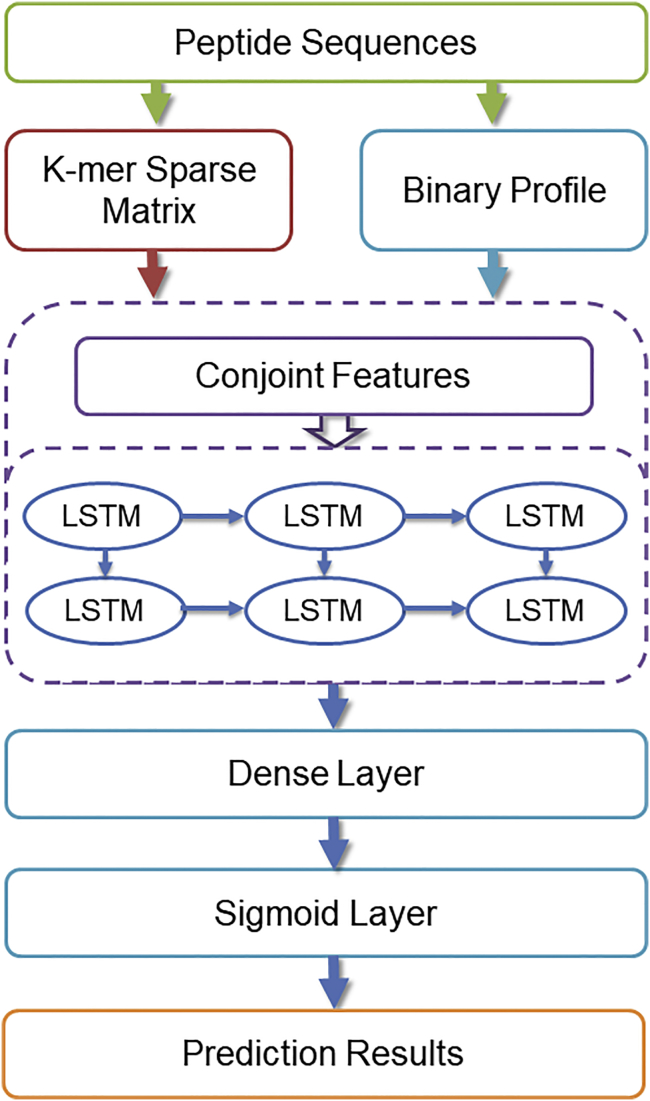
The Flowchat of Our ACP-DL Method We used the *k*-mer sparse matrix and binary profile feature to represent peptide sequences, and the deep LSTM model is trained to predict anticancer peptides.

To further evaluate the performance of our model, we evaluated the ACP-DL on two novel benchmark datasets. We also compared the purposed ACP-DL with existing state-of-the-art machine-learning models, e.g., SVM,^44^, ^45^ Random Forest (RF),^46^and Naive Bayes (NB).^47^ The 5-fold cross-validation experimental results showed that our method is suitable for the anticancer prediction mission with notable prediction performance. The workflow of ACP-DL is show in Figure 1.

## Results and Discussion

**Figure 2:**
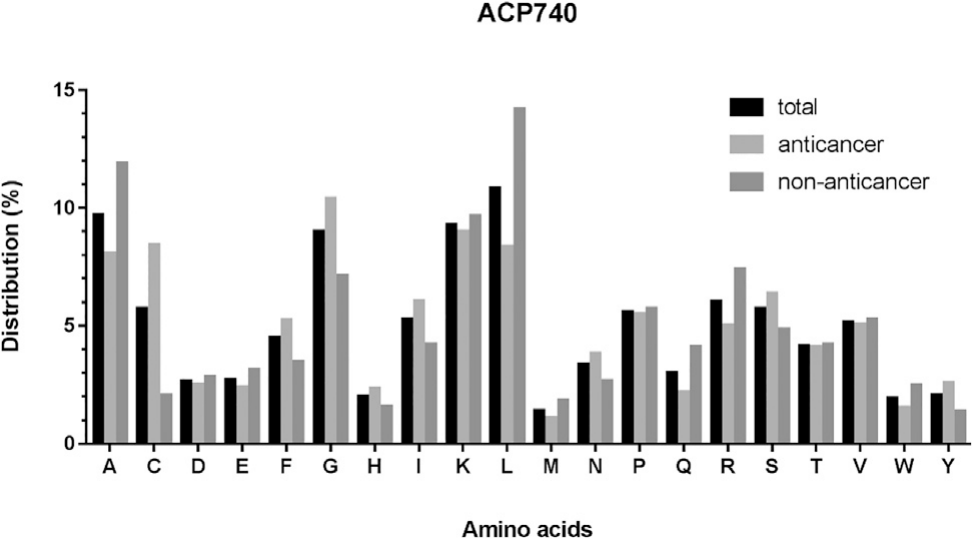
Comparison of Amino Acid Composition of Anticancer, Non-anticancer, and Total Peptides in Dataset ACP740

**Figure 3:**
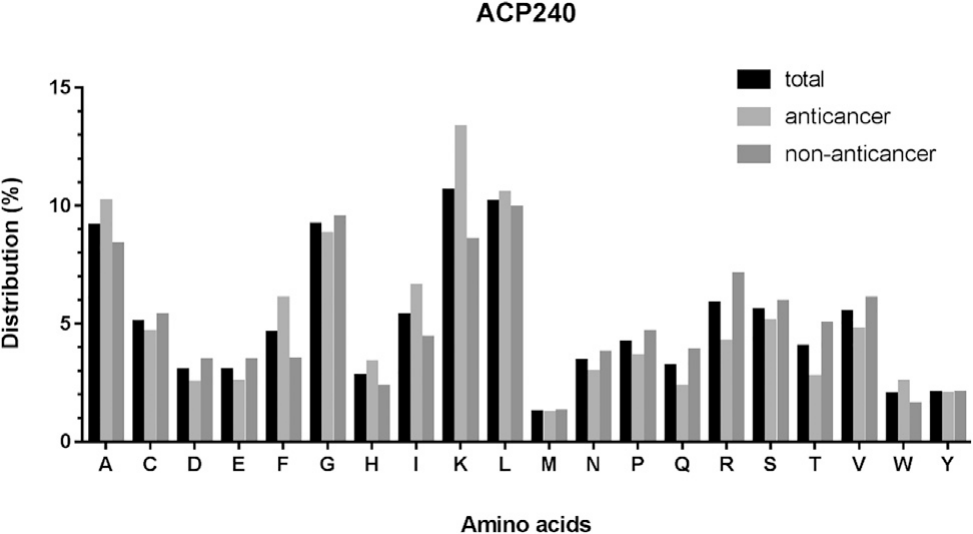
Comparison of Amino Acid Composition of Anticancer, Non-anticancer, and Total Peptides in Dataset ACP240

Above all, we compared the different distributions of amino acids in anticancer peptides, non-anticancer peptides, and all peptides in datasets ACP740 and ACP240. The results for ACP740 are shown in Figure 2, the composition of all 20 amino acids in these peptides were counted and compared. Certain residues, including Cys, Phe, Gly, His, Ile, Asn, Ser, and Tyr, were found to be abundant in anticancer peptides compared to non-anticancer peptides, while Glu, Leu, Met, Gln, Arg, and Trp were abundant in non-anticancer peptides compared to anticancer peptides. Similarly, as shown in Figure 3, in dataset ACP240, the Phe, His, Ile, and Lys were abundant in anticancer peptides. Since terminal residues play essential roles in biological functions of peptides.

### Evaluation of ACP-DL’s Capability to Predict Anticancer Peptides

First, we executed our model ACP-DL on the ACP740 and ACP240 datasets to evaluate its ability of predicting anticancer peptides. The 5-fold cross-validation details are offered in Tables 1 and 2.

**Table 1:** The 5-Fold Cross-Validation Details in the ACP740 Dataset

Fold Set	Acc (%)	Sens (%)	Spec (%)	Prec (%)	MCC (%)
1	79.73	81.94	77.63	81.94	59.58
2	83.11	85.71	80.00	86.30	66.39
3	81.08	79.75	84.00	78.08	62.22
4	85.81	86.49	85.33	86.30	71.63
5	77.70	79.17	76.00	79.45	55.47
Average	81.48 ± 3.12	82.61 ± 3.36	80.59 ± 4.01	82.41 ± 3.81	63.05 ± 6.23

**Table 2:** The 5-Fold Cross-Validation Details in the ACP240 Dataset

Fold Set	Acc (%)	Sens (%)	Spec (%)	Prec (%)	MCC (%)
1	93.75	89.66	99.99	86.36	87.99
2	81.25	77.42	92.31	68.18	63.02
3	87.50	88.46	88.46	86.36	74.83
4	83.33	90.91	76.92	90.91	67.83
5	81.25	76.67	92.00	69.57	63.53
Average	85.42	84.62	89.94	80.28	71.44

**Figure 4:**
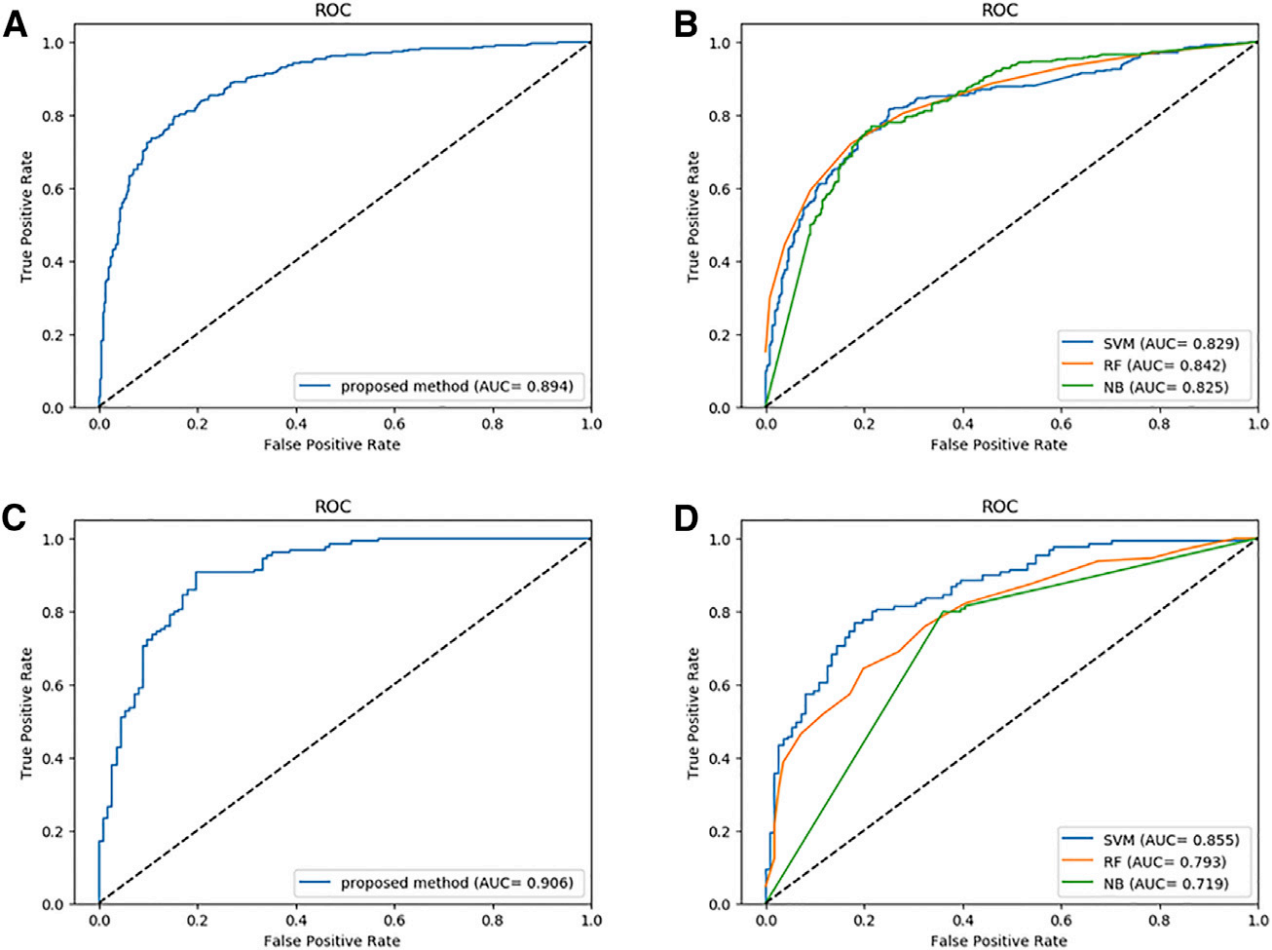
Performance of the Proposed Model ACP-DL and Comparison Model on Datasets ACP740 and ACP240 (A) The performance of the proposed model ACP-DL in dataset ACP740. (B) The performance of the comparison models in dataset ACP740, including SVM, RF, and NB. (C) The performance of the proposed model ACP-DL in dataset ACP240. (D) The performance of the comparison models in dataset ACP240, including SVM, RF, and NB.

The average accuracy of 5-fold cross-validation on ACP740 was 81.48% with 3.12% SD, the average sensitivity (Sens) was 82.61% with 3.36% SD, the average specificity (Spec) was 80.59% with 4.01% SD, the mean precision (Prec) was 82.41% with 3.81% SD, and the Matthews correlation coefficient (MCC) was 63.05% with 6.23% SD. ACP-DL showed an outstanding capability to identify anticancer peptides, performed an area under the receiver operating characteristic (ROC) curve (AUC) of 0.894, as shown in Figure 4A, and has achieved the best performance on the ACP740 dataset among all comparison methods.

The mean accuracy of 5-fold cross-validation on ACP240 was 85.42%, the average Sens was 84.62%, the average Spec was 89.94%, the mean Prec was 80.28%, and the MCC was 71.44%; and, the AUC of ACP-DL was 0.906, as shown in Figure 4C. In general, the performance of the deep learning model will become better with the increase in the scale of data, and the model that can achieve good results on smaller datasets will also achieve good results on huger data. Actually, the data scale of anticancer peptides is not very large, so we didn’t implement a neural network model with very complex architecture; and, to a certain extent, the 5-fold cross-validation is not conducive to the neural network model, because it further reduces the amount of training data. It is noteworthy that, although the dataset ACP240 was smaller than ACP740, our model ACP-DL still performed very well. The experimental results of rigorous cross-validation on benchmark dataset ACP740 and dataset ACP240 confirmed that our model has a good capability to predict anticancer peptides.

### Comparison with Three Widely Used Machine-Learning Models

**Figure 5:**
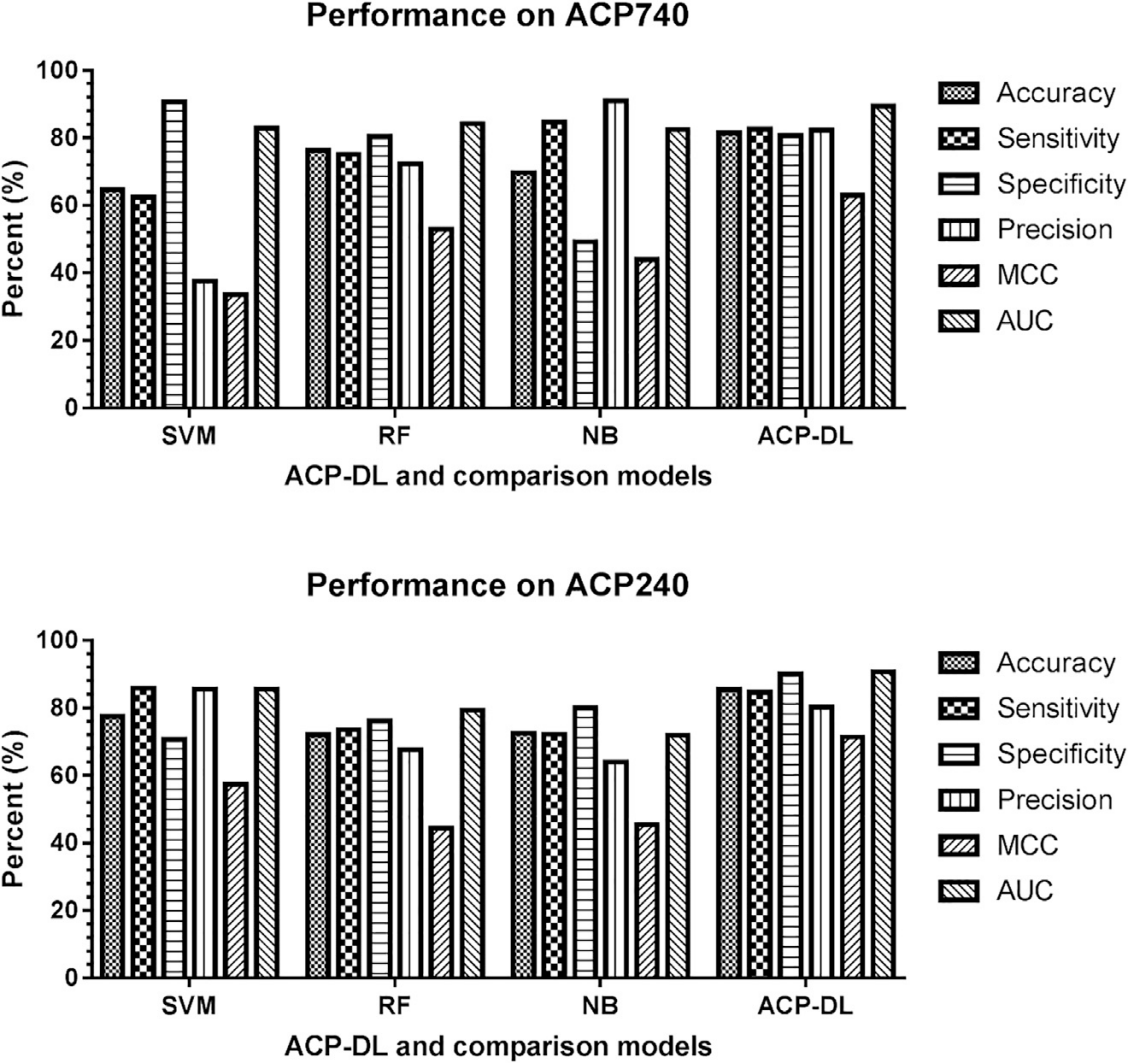
Comparison of SVM, Random Forest, Naive Bayes, and ACP-DL in Benchmark Datasets ACP740 and ACP240

To evaluate the ability of the purposed method, we further compared ACP-DL with other widely used machine-learning models on the same benchmark datasets, including ACP740 and ACP240. Here we have selected the SVM, RF, and NB models, and we built them using the same cross-validation datasets. The implementation of these three machine-learning models comes from Scikit-learn,^48^ and they were tested with default parameters. Since these methods were evaluated using the same evaluation criteria, the comparison model and deep learning model ACP-DL results are shown in Table 3 and Figures 4 and 5. ACP-DL obtained the most significant performance among the contrasted methods.

**Table 3:** Actual Performance of Comparison Models and ACP-DL in the Same Dataset

Dataset	Model	Acc (%)	Sens (%)	Spec (%)	Prec (%)	MCC (%)	AUC
ACP740	SVM	64.59	62.43	90.68^a^	37.57	33.57	0.829
	RF	76.35	75.10	80.34	72.27	53.06	0.842
	NB	69.73	84.70^a^	49.21	90.94^a^	43.98	0.825
	ACP-DL	81.48^a^	82.61	80.59	82.41	63.05^a^	0.894^a^
ACP240	SVM	77.50	85.89^a^	70.68	85.65^a^	57.31	0.855
	RF	72.08	73.53	76.09	67.63	44.38	0.793
	NB	72.50	72.26	79.94	63.95	45.44	0.719
	ACP-DL	85.42^a^	84.62	89.94^a^	80.28	71.44^a^	0.906^a^

a This measure of performance is the best among the compared methods.

Table 3 shows the details of the comparison. In the ACP740 dataset, our method ACP-DL significantly outperformed other methods with an accuracy of 81.48%, a Sens of 82.61%, a Spec of 80.59%, a Prec of 82.41%, an MCC of 63.05%, and an AUC of 0.894. ACP-DL increased the accuracy by over 5%, the MCC by over 10%, and the AUC by more than 5%, respectively. In the dataset ACP240, ACP-DL also performed remarkably with an accuracy of 85.42%, a Sens of 84.62%, a Spec of 89.94%, a Prec of 80.28%, an MCC of 71.44%, and an AUC of 0.906. ACP-DL improved the accuracy by over 8%, the Spec by over 10%, the MCC by over 14%, and the AUC by more than 5%, respectively. Obviously, the deep learning model shows its power, and our model is suitable for anticancer peptide identification and prediction. ACP-DL is a competitive model used to predict anticancer peptides and accelerate related research. The comparison experiment results proved our assumption.

### Conclusions

In this study, we proposed a deep learning LSTM model to predict potential anticancer peptides using high-efficiency feature representation. More specifically, we developed an efficient feature representation approach by integrating binary profile feature and *k*-mer sparse matrix of reduced amino acid alphabet feature to fully exploit peptide sequence information. Then we implemented a deep LSTM model to automatically learn how to identify anticancer peptides and non-anticancer peptides. To the best of our knowledge, this is the first time that the deep LSTM model has been applied to predict anticancer peptides.

Meanwhile, to evaluate the capability of the proposed method, we further compared ACP-DL with widely used machine-learning models in the same benchmark datasets, including ACP740 and ACP240; experimental results on the 5-fold cross-validation show that the proposed method achieved outstanding performance compared with existing methods, on benchmark dataset ACP740 with 81.48% accuracy at the AUC of 0.894 and on dataset ACP240 with an accuracy of 85.42% at the Spec of 89.94 and the AUC of 0.906, respectively. The improvement is mainly from the deep LSTM model’s model parameter optimization and effective feature representation from original peptide sequences. In addition, we have contributed two novel anticancer peptide benchmark datasets, ACP740 and ACP240, in this work.

It is anticipated that ACP-DL will become a very useful high-throughput and cost-effective tool, being widely used in anticancer peptide prediction as well as cancer research. Further, as demonstrated in a series of recent publications in developing new prediction methods,^49^, ^50^, ^51^ user-friendly and publicly accessible web servers will significantly enhance their impacts. It is our wish to be able to provide in the future a web server for the prediction method presented in this paper.

## Materials and Methods

In this study, we proposed a novel deep learning LSTM model to predict anticancer peptides, named ACP-DL, using high-efficiency features provided by *k*-mer sparse matrix and the binary profile feature. Furthermore, we evaluated ACP-DL’s predictive performance of anticancer peptides in benchmark datasets ACP740 and ACP240. Moreover, we compared ACP-DL with three widely used machine-learning models in the same datasets, including SVM,^44^ RF,^46^ and NB,^47^ to prove the robustness and effectiveness of the proposed method. Eventually, we made a summary, analysis, and discussion of the ACP-DL.

### Construction of Datasets

We constructed two novel benchmark datasets in this work for ACP identification, named ACP740 and ACP240. As previous studies suggested, the new datasets comprised both positive and negative datasets, while positive samples were experimentally validated ACPs and AMPs without anticancer function were collected as negative samples.

The positive anticancer peptide samples can be represented as $P^{+}$, and the negative non-anticancer peptides can be represented as $P^{-}$. So, the whole dataset can be represented as *P*.

(Equation 1) $$\text{P}=P^{+}\cup P^{-}$$

Moreover, there is no overlap between the positive and negative datasets.

(Equation 2) $$Ø=P^{+}\cap P^{-}$$

#### Dataset ACP740

We selected 388 samples as the initial positive data on the basis of Chen et al.’s^21^ and Wei et al.’s^24^ studies, of which 138 were from Chen et al.’s work and 250 were from Wei et al.’s work. Correspondingly, the initial negative data were 456 samples, of which 206 were from Chen et al.’s work and 250 were from Wei et al.’s work, respectively. To avoid the bias of dataset, the widely used tool CD-HIT^52^ was further used to remove those peptides sequences with a similarity of more than 90%. As a result, we finally obtained a dataset containing 740 samples, of which 376 were positive samples and 364 were negative samples.

#### Dataset ACP240

As the same procedure, to validate the generalization ability of the predictive model, we further constructed an additional dataset, named ACP240, which initially included 129 experimentally validated anticancer peptide samples as the positive dataset and 111 AMPs without anticancer functions as the negative dataset, respectively.

Moreover, those sequences with a similarity of more than 90% were removed using the popular tool CD-HIT.^52^ The similarity setting was consistent with previous studies.^21^, ^22^ The CD-HIT is available at http://weizhong-lab.ucsd.edu/cdhit-web-server. There was no overlap between dataset ACP740 and dataset ACP240, and these two datasets are both non-redundant datasets. The two benchmark datasets are publicly available at https://github.com/haichengyi/ACP-DL.

### Representation of the Peptide Sequences

A peptide sequence can be represented as follows:

(Equation 3) $$\text{P}=p_{1}p_{2}p_{3}p_{\ldots }p_{l},$$

where $p_{1}$ represents the first residue in the peptide P, $p_{2}$ denotes the second residue in the peptide P, and so on; *l* represents the length of P. Note that the residue $p_{i}$ is an element of the standard amino acid alphabet to train a machine-learning model; the first step is to convert diverse-length peptides into fixed-length feature vectors. In this study, we exploited two feature representation methods, as described below.

#### Binary Profile Feature (BPF)

As mentioned above, there are 20 different amino acids in the standard amino acid alphabet (A, C, D, E, F, G, H, I, K, L, M, N, P, Q, R, S, T, V, W, and Y). Each amino acid type is encoded with the following feature vector composed of 0/1. More specifically, the first amino acid type A in the alphabet is encoded as f(A) = (1,0,…,0), the second amino acid type C is encoded as f(C) = (0,1,…,0), and so on. Subsequently, for a given peptide sequence P, its N terminus with the length of *k* amino acids was encoded as the following feature vector:

(Equation 4) $$\text{BPF}\left(\text{k}\right)=\left[f\left(p_{1}\right),f\left(p_{2}\right),\ldots f\left(p_{k}\right)\right],$$

where *k* represents the length of the N terminus of the peptide P.^22^ Thus, the dimension of BPF(P) is 1 × 20. Experiments show that the best results can be achieved when *k* is set to 7. So, one given peptide sequence is encoded to a 1×140 feature vector by binary profile.

#### K-mer Sparse Matrix

We also encoded the peptide sequence by using the *k*-mer sparse matrix previously proposed.^41^ In detail, its *k-*1 consecutive nucleotides and *k* consecutive nucleotides are regarded as a unit. 3-mer of peptides is composed of 3 amino acids.^53^ First the 20 amino acids were reduced into 7 groups based on their dipole moments and side chain volume: Ala, Gly, and Val; Ile, Leu, Phe, and Pro; Tyr, Met, Thr, and Ser; His, Asn, Gln, and Tpr; Arg and Lys; Asp and Glu; and Cys.^16^, ^54^, ^55^ So, the peptide sequence was reduced into a 7-letter alphabet. Then we scanned each peptide sequence from left to right, stepping one amino acid at a time, which is considered the characteristics of each amino acid.

Suppose an above-mentioned peptide sequence length is *L*, there would be $7^{k}$ different possible *k*-mer and an L−k+1 step appearing in the RNA sequence.

One peptide sequence is transformed into a $7^{k}\times \left(L-k+1\right)$ *k*-mer sparse matrix *M*. Initialization of all elements is 0. When $m_{j}m_{j+1}m_{j+2}$ are just equal to the *i*_th_ *k*-mer among $7^{k}$ different *k*-mer, set the element *a*_*ij*_ *=* 1. The rest can be handled in the same way. Thus, an input peptide sequence is converted into a $7^{k}\times \left(L-k+1\right)$ matrix *M*.

In this study, the value of *k* is set to 3 to process the peptide sequence. The *k*-mer sparse matrix *M* can be defined as follows:

(Equation 5) $$\text{M}={\left({\text{a}}_{\text{ij}}\right)}_{7^{\text{k}}}\times \left(\text{L}-\text{k}+1\right)$$

(Equation 6) $$a_{ij}=\{\begin{array}{c} 1,\;if\;{\text{m}}_{\text{j}}{\text{m}}_{\text{j}+1}{\text{m}}_{\text{j}+2}=k-mer\left(i\right)\\ 0,\;else \end{array}.$$

The *k*-mer sparse matrix *M* is a low-rank matrix, which almost retained all the raw information, including sequence frequency, position, and order hidden information. Then, singular value decomposition (SVD)^56^ is used to reduce one two-dimensional matrix *M* into a 1×343 feature vector.

Finally, we conjoined two different feature representation methods’ output, each peptide sequence gain 1×483 conjoined feature vector. Meanwhile, the whole dataset was transformed as a 2D matrix here. The feature matrix was reshaped into a 3D tensor for training the LSTM model, while the feature matrix without being formally reshaped was used to train the comparison model.

### Deep LSTM Model Architecture

LSTM is an improvement of a recurrent neural network (RNN), which is mainly used in the natural language processing (NLP) and speech recognition field.^57^, ^58^, ^59^ Different from a traditional neural network, an RNN can take advantage of sequence information. Theoretically, it can utilize the information of arbitrary length sequence; but, because of the problem of vanishing gradient in the network structure, it can only retrospectively utilize the information on time steps that are close to it in practical applications. To solve this problem, LSTM was presented with specially designed network architecture, which can learn long-term dependency information naturally. A general architecture of LSTM is composed of an input gate, a forget gate, an update gate, and a memory block. The improvement of LSTM is mainly from incorporating a memory cell that accepts the network to learn when to forget previous hidden states and when to update hidden states, according to the input information through time. It uses dedicated storage units to store information. To our knowledge, the deep LSTM model was first applied to predict novel anticancer peptides in this work.

LSTM selectively passes information through a gate unit, which mainly is by means of a sigmoid neural layer and a dot multiplication operation. Each element of the sigmoid layer output (a vector) is a real number between 0 and 1, representing the weight (or percentage) that the corresponding information passes through. For example, 0 means no information is allowed, and 1 means let all information pass.

#### Forget Gate

In the information flow processing of LSTM, the first step is to decide what information will discord from the cell state. This decision is accomplished by a way known as forget gate. Forget gate reads $h_{t-1}$ and $x_{t}$, then outputs a value between 0 and 1 for each digit in cell state $C_{t-1}$; 1 means reserved absolutely and 0 means discard completely.

(Equation 7) $$f_{t}=\sigma \left(W_{f}\cdot \left[h_{t-1},x_{t}\right]+b_{f}\right)$$

Here, the $h_{t-1}$ represents the output of the previous cell, $x_{t}$ represents the current cell input, and σ means Sigmoid function.

#### Input Gate

The next step is to decide how much new information will be added to the cell state. To do this, there are two steps: first, a Sigmoid layer called the input gate layer determines which information needs to be updated; and then, a tanh layer generates a vector, which is the alternate content ${\tilde{C}}_{t}$ to update. We combined the two parts to update the state of cell.

(Equation 8) $$i_{t}=\sigma \left(W_{i}\cdot \left[h_{t-1},x_{t}\right]+b_{i}\right)$$

(Equation 9) $${\tilde{C}}_{t}=\text{tanh}\left(W_{c}\cdot \left[h_{t-1},x_{t}\right]+b_{c}\right)$$

(Equation 10) $$C_{t}=f_{t}*C_{t-1}+i_{t}*{\tilde{C}}_{t}$$

We multiply the old state with $f_{t}$ and discard the information we need to discard. Then we add $i_{t}*{\tilde{C}}_{t}$. This is the new candidate value, which is changed according to the degree of each state we decide to update.

#### Output Gate

Ultimately, we need to determine what output is. This output will be based on our cell state, but it is also a filtered version. First, we run a sigmoid layer to determine which part of the cell state will be exported. Then, we process the cell state through a tanh function (to get a value between −1 and 1) and multiply it with the output of the Sigmoid gate, and eventually we just output the portion of the output we determine.

(Equation 11) $$o_{t}=\sigma \left(W_{o}\cdot \left[h_{t-1},x_{t}\right]+b_{o}\right)$$

(Equation 12) $$h_{t}=o_{t}*\tanh\left(C_{t}\right)$$

In this experiment, considering the limited scale of anticancer peptide samples, we set the parameter of LSTM input layer to 128, and the output of LSTM layers was fed into a dense layer (a fully connected neural network layer) as input to obtain a final prediction result. We also used a sigmoid function as an activation function in the proposed model. The mathematical behaviors of a sigmoid function can be demonstrated as follows:

(Equation 13) $$\text{σ}=\text{sigmoid}\left(x\right)=\frac{1}{\left(1+e^{-x}\right)}.$$

Between them, the dropout layer was applied to reduce over-fitting and enhance the neural network model robustness, and the parameter *dropout* was set to 0.25. Moreover, a loss function can measure the performance of machine-learning models. We selected to use log loss function (binary cross-entropy) corresponding to sigmoid function as loss function, which can be defined as:

(Equation 14) logloss(t,p)=−((1−p)×log(1−p)+t×log(p)),

where *p* and *t* represent the prediction output of model and true target value, respectively. Finally, the Adam^60^ optimizer was used to update the weights of network iteratively, which is popular in the deep learning field and combined the advantage of root-mean-square propagation (RMSProp) and adaptive gradient (AdaGrad) algorithm.

The implementation of the deep learning model is based on the Keras framework, which is capable of running on top of TensorFlow, Theano, or CNTK and is supported on both GPUs and CPUs. It was developed with a focus on enabling fast experimentation.^61^

### Performance Evaluation Criteria

In this study, we proposed a novel deep learning LSTM model, ACP-DL, using an efficiency feature to predict potential anticancer peptides. We used 5-fold cross-validation to evaluate the performance of ACP-DL and comparison models. In each validation, all data randomly divide into five equal parts: the 4-fold set data are taken as training data, and the remaining 1-fold data are taken as test data. To guarantee the unbiased comparison, it was confirmed that there was no overlap between training data and test data. The final validation result was the average of 5-fold with SDs. We followed the widely used evaluation criteria,^62^, ^63^ including accuracy (Acc), Sens or recall, Spec, Prec, and MCC, defined as follows:

(Equation 15) $$\text{Acc}=\frac{TN+TP}{TN+TP+FN+FP}$$

(Equation 16) $$\text{Sens}=\frac{TP}{TP+FN}$$

(Equation 17) $$\text{Spec}=\frac{TN}{TN+FP}$$

(Equation 18) $$\text{Prec}=\frac{TP}{TP+FP}$$

(Equation 19) $$\text{MCC}=\frac{TP\times TN-FP\times FN}{\sqrt{\left(TP+FP\right)\left(TP+FN\right)\left(TN+FP\right)\left(TN+FN\right)}},$$

where *TN* indicates the true negative number, *TP* denotes the true positive number, *FN* represents the false negative number, and *FP* stands for the false positive number. Certainly, the ROC curve and the AUC were also adopted to evaluate the performance.

## Author Contributions

H.-C.Y. and Z.-H.Y. conceived the algorithm, carried out analyses, prepared the datasets, carried out experiments, and wrote the manuscript. Other authors designed, performed, and analyzed experiments and wrote the manuscript. All authors read and approved the final manuscript.

## Conflicts of Interest

The authors declare no competing interests.
